# Prevalence, risk-factors, and antimicrobial susceptibility profile of methicillin-resistant Staphylococcus aureus (MRSA) obtained from nares of patients and staff of Sokoto state-owned hospitals in Nigeria

**DOI:** 10.3205/dgkh000360

**Published:** 2020-10-12

**Authors:** Shuaibu Suleiman Adeiza, Josiah Ademola Onaolapo, Busayo Olalekan Olayinka

**Affiliations:** 1 Department of Pharmaceutical Microbiology, Faculty of Pharmaceutical sciences, Ahmadu Bello University, Zaria, Kaduna, Nigeria

**Keywords:** Staphylococcus aureus, dendrogram, antimicrobial resistance, methicillin resistance, regression

## Abstract

**Aim:** The aim of the study was to determine the prevalence of methicillin-resistant *Staphylococcus aureus* (MRSA) obtained from the nasal cavity of participants and investigate the antibiotic resistance profiles of the isolates from Sokoto state, Nigeria.

**Methods:** Nasal swabs of both nares were obtained from 378 participants across three study centers within the six-month study period. The *Staphylococcus aureus* isolates recovered were characterized, and their resistance phenotype determined in conjunction with MRSA prevalence.

**Results:** Phenotypic screening of isolates obtained in this study revealed a total of 131 (17.3%) coagulase-positive Staphylococci out of 756 samples. Of this number, there were 81 (61.8%) *S. aureus*, 36 (27.5%) *Staphylococcus intermedius*, 6 (4.5%) *Staphylococcus hyicus,* and 8 (6.1%) *Staphylococcus schleiferi*.

**Conclusion:** This study found a prevalence of 61.8% and 46.9% of *S. aureus* and MRSA among the studied hospitals in Sokoto state, thus demonstrating that the nares of the hospital populace are not free from *S. aureus* and MRSA colonization.

## Zusammenfassung

**Ziel:** Ziel der Studie war es, die Prävalenz von Methicillin-resistenten *Staphylococcus aureus* (MRSA) aus der Nasenhöhle der Teilnehmer zu bestimmen und die Antibiotika-Resistenz-Profile der Isolate aus dem Bundesstaat Sokoto, Nigeria, zu untersuchen.

**Methode:** Von 378 Teilnehmern aus drei Studienzentren wurden im Verlauf von 6 Monaten Abstriche aus beiden Nares entnommen. Die gewonnenen *Staphylococcus aureus*-Isolate wurden charakterisiert und ihr Resistenz-Phänotyp in Verbindung mit der MRSA-Prävalenz bestimmt.

**Ergebnisse:** Das phänotypische Screening der Isolate ergab 131 Koagulase-positive Staphylokokken, davon 81 *Staphylococcus aureus* (61,8%), 36 *Staphylococcus intermedius* (27,5%), 6 *Staphylococcus hyicus* (4,5%) und 8 *Staphylococcus schleiferi* (6,1%).

**Schlussfolgerung:** Die Studie zeigt eine relative Prävalenzrate von 61,8% und 46,9% von *S. aureus* und MRSA unter den untersuchten Krankenhäusern im Bundesstaat Sokoto, was darauf schließen lässt, dass die Nasen der Krankenhausbevölkerung nicht frei von *S. aureus* und MRSA-Kolonisation sind.

## Introduction

*Staphylococcus aureus* is a major cause of morbidity and mortality globally, both within the community and in healthcare settings [1]. Its ability to cause disease is enhanced by its virulence factors and resistance to antibiotics used in its treatment, exemplified by the advent of methicillin-resistant *Staphylococcus aureus* (MRSA). Most MRSA strains harbor *mecA* gene on a staphylococcal cassette chromosome mec (SCCmec) and codes for a modified penicillin binding protein (PBP2a). This protein has a reduced affinity for all beta-lactam and Betalactam/beta-lactamase inhibitor combination antibiotics [2].

The burden of MRSA is mostly less than 50% in most of the African countries. In Tunisia, MRSA prevalence is from 16% to 41%, South Africa 24% to 36%, Botswana 23%–44%, Egypt 45% and 52%, Libya 31%) and Nigeria 55% (Northern) and 39% (Southern) [3], [4], [5]. This clear variation might be due to different environmental factors, strain diversity and differences in antibiotics usage. In Kenya, there is a noticeable difference in MRSA prevalence reported in clinical isolates; one study [6] reported a prevalence rate of about 3.7%. while another reported 87.2% [7].

A previous study showed that *S. aureus* clones obtained from nasal specimens of septicemic patients were identical to those from blood isolates (≈82%), thus evincing the relationship between nasal carriage and infection. Healthy carriers may transmit the organism to other members of the community or immunocompromised persons [8]. 

With this study, we intended to fill in the knowledge gap about resistance trends of MRSA both in healthcare and community settings in Sokoto, North West Nigeria. The results of the present study will facilitate implementation of strategies for the prevention and effective management of methicillin-resistant infections in Sokoto, Nigeria. 

This study examines the prevalence of methicillin resistant *Staphylococcus aureus* (MRSA) obtained from the nasal cavity of study participants and explores the antibiotic resistance profiles of isolates from Sokoto state-owned hospitals.

## Material and methods

### Study area

This work was carried out in Sokoto State, in the extreme north-west of Nigeria, between longtitudes 4°8¹ and 6°54¹ and latitude 12°N and 13°58¹N. The state includes twenty-three (23) Local Government areas and shares borders with Niger Republic to the north and north-east, Kebbi state, to the west and south-east, and Zamfara state, to the east. The State covers an area of 32,000 km^2^ [9]. The warmest months are February to April, when daytime temperatures can exceed 45°C. The State has a population of 3,696,999 [10]. There are two major ethnic groups, Hausa and Fulani. The majority of the populace are agriculturists, although trades such as blacksmithing, dyeing and leather tanning are also practiced [11].

### Ethical consideration 

Ethical clearance was obtained from the ethics review boards of Sokoto State Ministry of Health and three hospitals spread across three local government areas in the state (Maryam Abacha Women’s and Children’s Hospital, Specialist Hospital and Orthopedic Hospital Wamakko) after approval of the research protocol (Approval number=SMH/1580/V. IV).

### Informed consent

The participants in the study gave informed consent following detailed (oral) explanation of the study and assurance of anonymity. 

### Sample size determination

The sample size was determined using the formula specified by Thrusfield et al. [12] by taking a previous prevalence of *S. aureus*, 40.6% [13], found in a study carried out to establish the prevalence and nasal carriage of MRSA among adult patients in Cameroon. Accordingly, the calculated value for sample size equaled 232 samples. 

### Exclusion criteria

We excluded participants with nasal anatomical deformities or bleeding nostrils from the study. 

### Specimen collection

**Figure 1:**
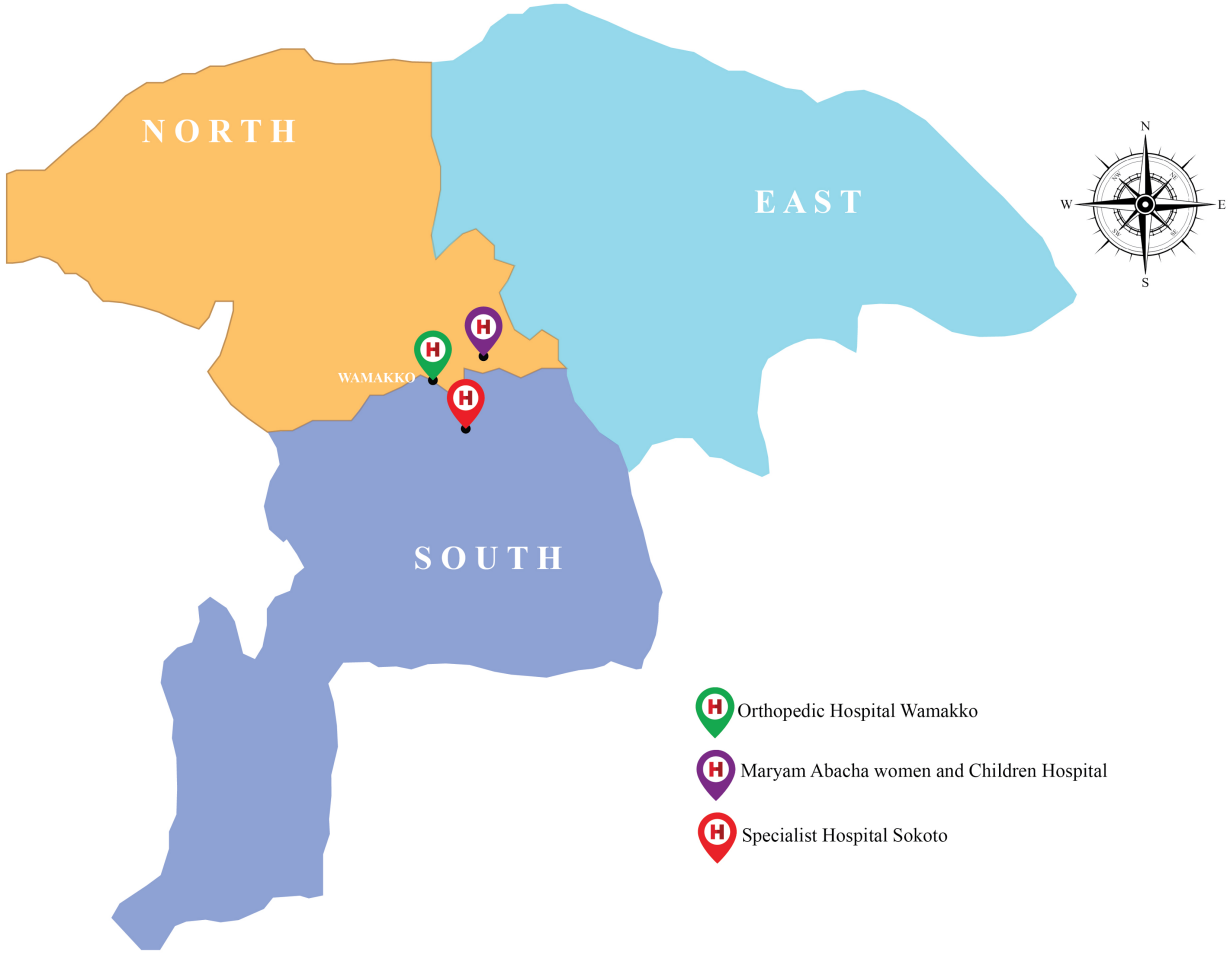
Map of Sokoto State showing Maryam Abacha Women’s and Children’s Hospital (purple: Sokoto North) and Specialist Hospital (red: Sokoto South), Orthopedic Hospital Wamakko (green)

Three health-care centers from three of the largest local government areas (LGA) in Sokoto state, specifically Sokoto South (Specialist Hospital Sokoto), Sokoto North (Maryam Abacha Women’s and Children’s Hospital Sokoto) and Wamakko (Orthopedic Hospital) LGA were selected for this study (Figure 1 (Fig. 1)). 

Nasal swabs were collected randomly from healthcare workers, inpatients, outpatients, security guards and janitors. A structured proforma pertaining to demography and risk factors for* S. aureus* and MRSA colonization was discussed with participants (voluntarily) before sample collection. Samples were collected only from participants who gave informed consent. 

The right and left nostrils of consenting participants were swabbed using two different commercially available swab sticks. The swab was introduced into each nostril (pre-moistened with sterile normal saline) by rolling it 4 to 5 times (clockwise and anticlockwise) against the nostril wall [14]. Collected samples were then placed in a tube containing aqueous peptone solution, labeled, stored in icepacks and transported immediately to the central research laboratory for further processing.

### Phenotypic screening to identify Staphylococcus aureus

Upon arrival in the laboratory, nasal swabs were inoculated into mannitol salt agar and incubated for 18–24 hours at 37°C. Characteristic golden yellow colonies with yellowish background emerging from the overnight culture were noted as presumptive *Staphylococcus aureus* colonies. The colonies were further characterized by standard bacteriological procedures such as colony morphology, Gram reaction, catalase test, coagulase test and Microgen™ STAPH-ID kit [15]. The phenotypically characterized isolates were then inoculated into nutrient agar slants and stored until further characterization.

### Antibiotic susceptibility testing (AST)

Phenotypically confirmed isolates of *Staphylococcus aureus* were suspended in brain-heart infusion broth and incubated at 35°C for 7 hours to attain a 0.5 MacFarland standard. The suspension was transferred into a glass cuvette, and the absorbance at 630 nm was determined and adjusted to 0.1. With the aid of a mono-channel micropipette, 1000 µl of the standardized inoculum was introduced into Mueller-Hinton agar (MHA) and spread throughout the media by means of a sterile glass rod and allowed to dry. Subsequently, the antimicrobial disks were placed aseptically using sterile forceps, allowed a pre-diffusion time of 1 hour and incubated for 24 hours at 37°C [16].

Antimicrobial susceptibility testing was performed by Kirby-Bauer disk diffusion on Mueller Hinton medium, according to Clinical and Laboratory Standards Institute standards, using antibiotic disks representative of ten antibiotic classes: clindamycin (2 µg), erythromycin (15 µg), ceftazidime (30 µg), trimethoprim/sulfamethoxazole (1.25/23.75 µg), chloramphenicol (30 µg), linezolid (30 µg), tetracycline (30 µg), cefoxitin (30 µg), levofloxacin (5 µg), gentamicin (120 µg). The disks were placed 30 mm apart. After the disks were applied onto the agar surface, the plates were incubated at 35°C for 24 hours [16]. Zone of inhibition diameters were measured and entered real-time into the WHONET 5.6 software database, which then interpreted the results using CLSI 2018 break-points built into the system. The results were recorded as interpreted values, i.e., “R” (Resistant), “I” (Intermediate) and “S” (Sensitive) [17].

Multi-resistant strains were divided into MDR (multiple drug-resistant), XDR (extensively drug-resistant) and PDR (pan drug-resistant). MDR bacteria were defined as resistant to antibiotics in at least three different classes of antibiotics. XDR bacteria were characterized by their sensitivity to only three or fewer classes of antibiotics, and the PDR bacteria were resistant to all classes of antibiotics used in the study [18].

### Oxacillin resistance screening agar base (ORSAB) test

A standardized suspension (0.5 McFarland) of *S. aureus* isolates was prepared and inoculated onto ORSAB medium pre-supplemented with 6 µg/ml oxacillin and 4% sodium chloride (ORSAB) to form a microbial lawn, which was incubated at 35°C for 24 hours. The emergent bluish colonies from the overnight cultures were considered methicillin resistant [19], [20].

### Latex slide agglutination test for detec- tion of penicillin-binding protein (PBP2a)

Penicillin binding protein (PBP2a) latex agglutination test kit (Oxoid, UK) is a rapid latex agglutination test kit used to confirm successful isolation of MRSA by the detection of PBP2a in *Staphylococcus isolates*. The presence of PBP2a was determined according to the guideline provided by the manufacturer. Colonies from ORSAB medium symbolic of methicillin resistance were tested for the production of the PBP2a protein. The protein from resistant bacteria was extracted using two extraction reagents (DR0903 and DR0904). A loopful of organisms was placed into a 1.5 ml microcentrifuge tube with 4 drops of extraction reagent DR0903 and placed in a water bath set at 75°C. After 5 minutes, the tubes were removed and allowed to cool. A single drop of extraction reagent DR0904 was added to each tube, mixed well, and centrifuged at 3,000 rpm for five minutes. The supernatant was used for the test. The latex agglutination test was conducted on test cards provided with the kit [21]. 

The sample number was written in one circle and the other circle was labeled “control”. The latex reagents were well mixed, and a drop of test latex (DR0901) or control latex (DR0902) was added to their respectively labeled circles on the test card. 50 µL of the supernatant from the specimen was placed on the test circle and mixed with the mixing stick provided. The card was picked up and rocked back and forth for about three minutes while looking for agglutination under normal ambient light. The outcome of the test and the control reactions were noted visually. Agglutination of the test but not the control latex was considered positive, while no agglutination was considered negative [22].

### Statistical analysis

The data were entered using SPSS version 25, their completeness and clearance were checked and then transferred to SAS, version 9.3 (SAS Institute Inc., Cary, North Carolina, USA). All the data were expressed in numbers and frequencies. Descriptive statistics was performed on the presence/absence of *S. aureus* and MRSA within the nares of each individual over the study period to determine the rate of carriage. 

### Modelling

**Figure 2:**
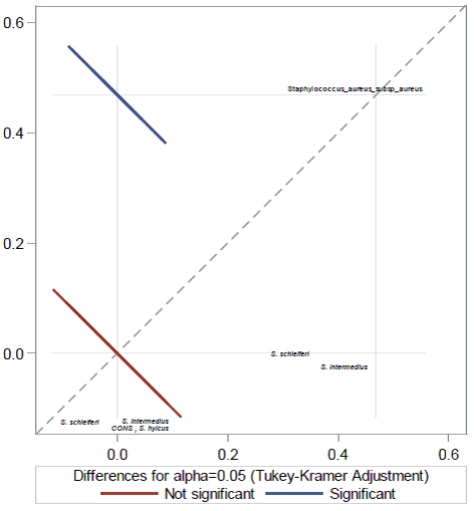
One-way ANOVA relationship plot of MRSA with all coagulase-positive staphylococcal isolates

Univariate associations of each risk factor with *S. aureus* and MRSA was examined using the frequency procedure of SAS, and a p-value ≤0.2 was considered significant. The risk factors found to be statistically significant by univariate logistic regression analysis at p≤0.2 were further evaluated by multivariate (forward selection procedure) regression model (p≤0.05) for associations with *S. aureus* and other coagulase positive staphylococci. A binary logistic regression model was used determine the association of the risk factor with MRSA. and p≤0.05 at a 95% CI was considered statistically significant. By using regression models, Adjusted Odds ratios (AOR) and 95% CI were calculated to identify how variables associated independently with the development of MRSA or *S. aureus* colonization. The primary analysis finally compared participants in whom MRSA colonization had occurred with those without MRSA colonization using one-way analysis of variance (ANOVA) (Figure 2 (Fig. 2)). The factors that fit the model appeared the most statistically plausible.

## Results

**Figure 3:**
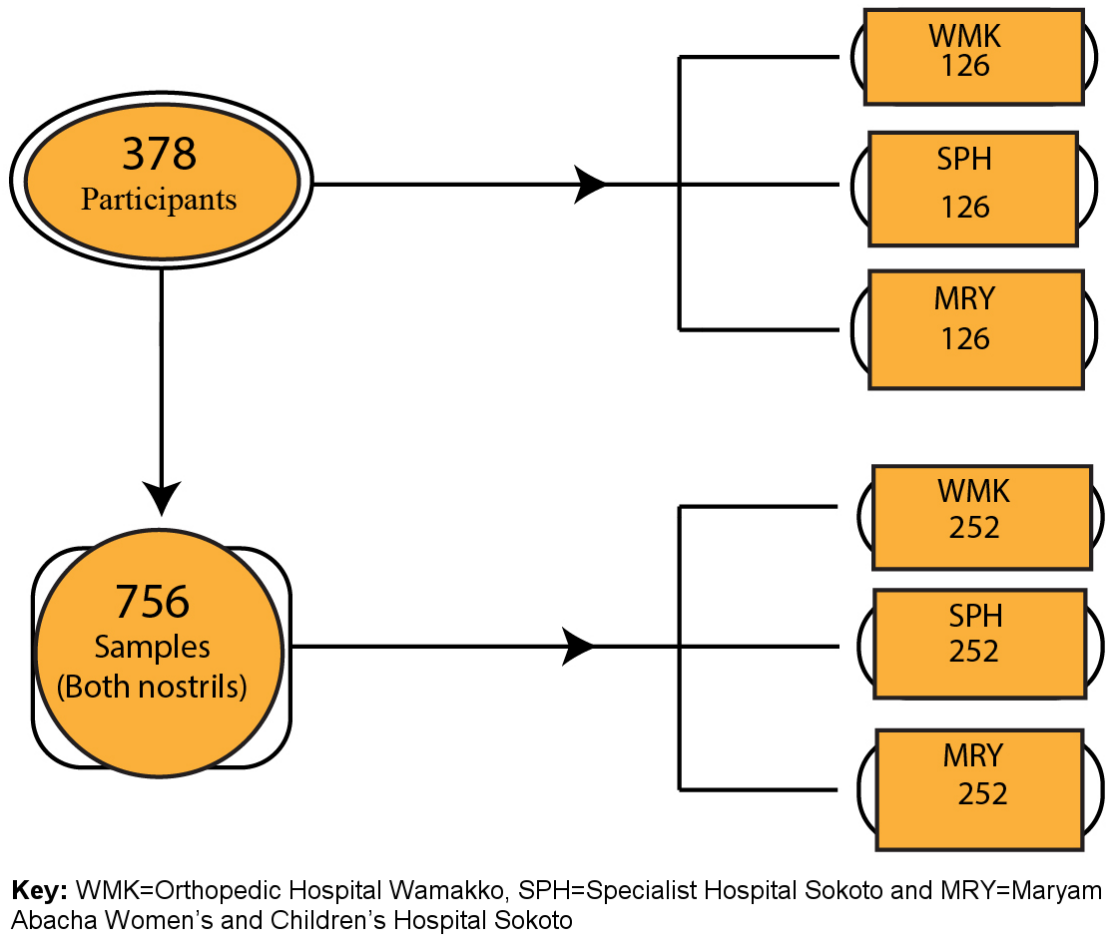
Schematic representation of nasal sampling plan employed in this study

A total of 756 nasal samples were obtained from 378 participants across three study centers within the six-month (February 2018 to July 2018) study period (Figure 3 (Fig. 3)). Briefly, 202 samples from healthcare workers (HCW), 214 from outpatients, 148 from inpatients, 108 from security personnel and 84 from janitors. A detailed summary of study participants is presented in Table 1 (Tab. 1). Phenotypic screening of isolates obtained in this study revealed a total of 131 (17.3%) coagulase-positive staphylococci out of 756 samples. Of this number, there were 81 (61.8%) S*. aureus*, 36 (27.5%) *Staphylococcus intermedius*, 6 (4.5%) *Staphylococcus hyicus* and 8 (6.1%) *Staphylococcus schleiferi*. The percentage carriage of *S. aureus* among participants in Specialist Hospital Sokoto was the highest (13.1%) compared with other hospitals in the study. Overall, healthcare workers were the participants with the highest carriage rate (12.9%).

**Figure 4:**
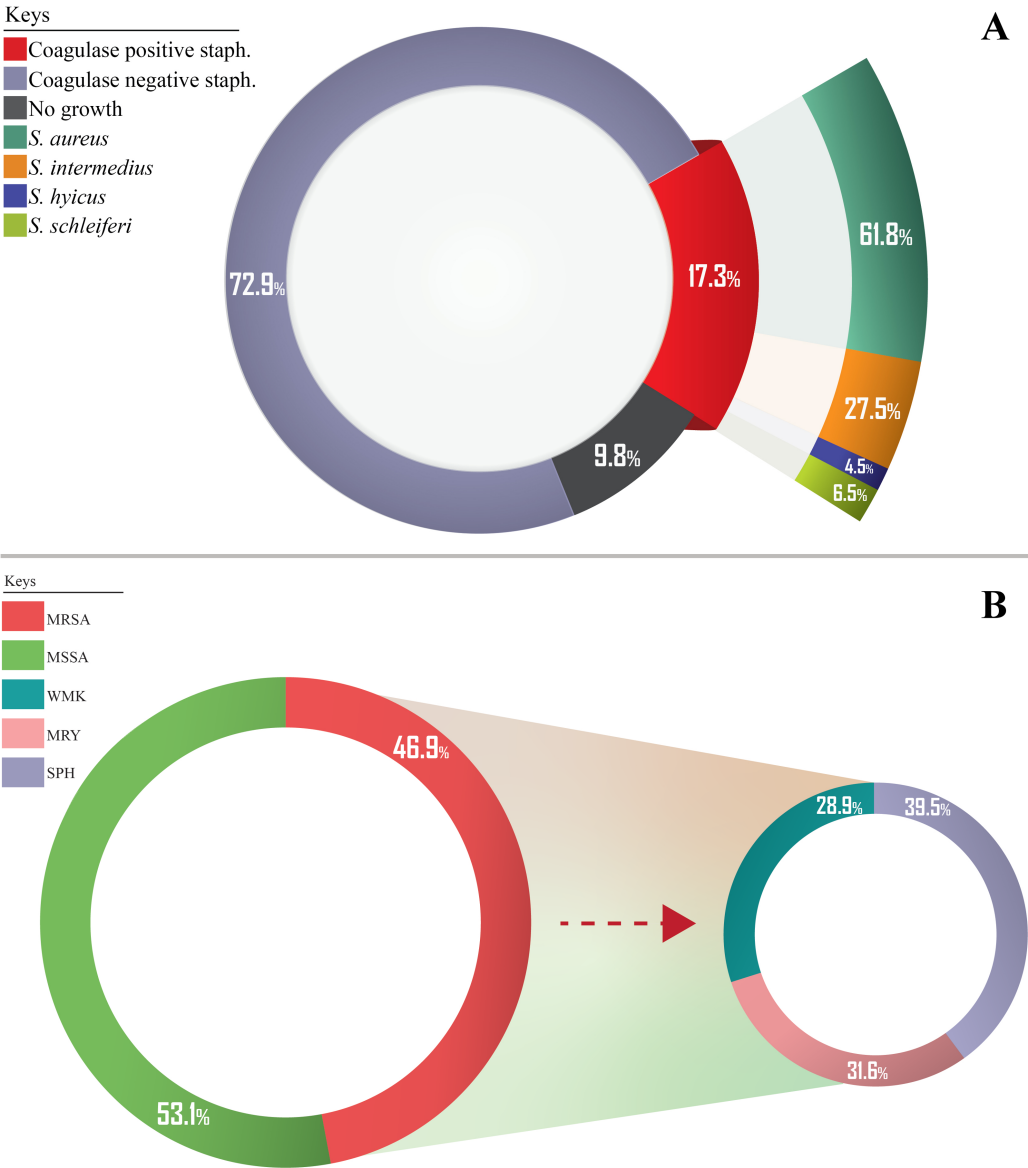
(A) Distribution of coagulase-positive and -negative staphylococcal isolates; (B) Percentage distribution of MRSA among phenotypically confirmed *S. aureus* at various study centers

Figure 4 (Fig. 4) and Table 2 (Tab. 2) show the carriage rate of coagulase-positive and -negative staphylococcal isolates. Carriage of *S. aureus* was higher in the right nostril with a prevalence rate of 63% (95%, CI: 37.7–57.1) compared to the 37% (95%, CI: 20.2–42.8) rate in the left nostril. Among the study centers, Specialist Hospital Sokoto had the highest prevalence rate of 40.7% (95% CI: 22.7–46.3). Carriage of *S. aureus* was highest among healthcare workers with a prevalence rate of 32.1% (95% CI: 17.0–38.1).

Table 3 (Tab. 3) shows the distribution of coagulase-negative and -positive staphylococcal isolates by risk factor. In the table, the percentage carriage of *S. aureus* is higher among the female participants, 59.3% (95% CI: 35.4–63.6). The rates were also higher among married participants 50.6% (95% CI: 36.3–68.7) than single ones 49.4% (95% CI: 35.3–67.3). The highest prevalence rates occurred among the age group 21–25 (34.6% [95% CI: 18.6–40.4]). According to antibiotic usage, the results obtained showed a high prevalence rate of *S. aureus* among participants who had a recent history (2 months) of antibiotic use (80.2% [95% CI: 50.2–82.9]). Table 3 (Tab. 3) also shows that lower prevalence rates of *S. aureus* colonization were found among participants who were diagnosed as HIV positive (18.5% [95% CI: 7.0–38.7]). Regarding tobacco usage, this study found that the colonization rate of *S. aureus* was higher among non-tobacco users (60.5% [95% CI: 36.2–64.8]). A lower prevalence rate of *S. aureus* colonization was also documented among participants who were diagnosed as HIV positive (18.5% [95% CI; 7.0–38.7]). Furthermore, this study also found that participants with diabetes and renal disease who were *S. aureus* carriers did not show a high prevalence rate (44.4% [95% CI: 25.2–49.8]) and (6.2% [95% CI: 1.6–11.6], respectively), when compared with non-carrier participants without diabetes or renal disease. Figure 4B (Fig. 4) illustrates that most of the recovered coagulase-positive staphylococcal isolates were from participants at Specialist Hospital Sokoto. These isolates included *S. aureus* with 33 (40.7%), *S. intermedius* with 13 (36.1%), *S. hyicus* with 4 (66.7%), and *S. schleiferi* with 3 (37.5%) of their discrete totals.

**Figure 5:**
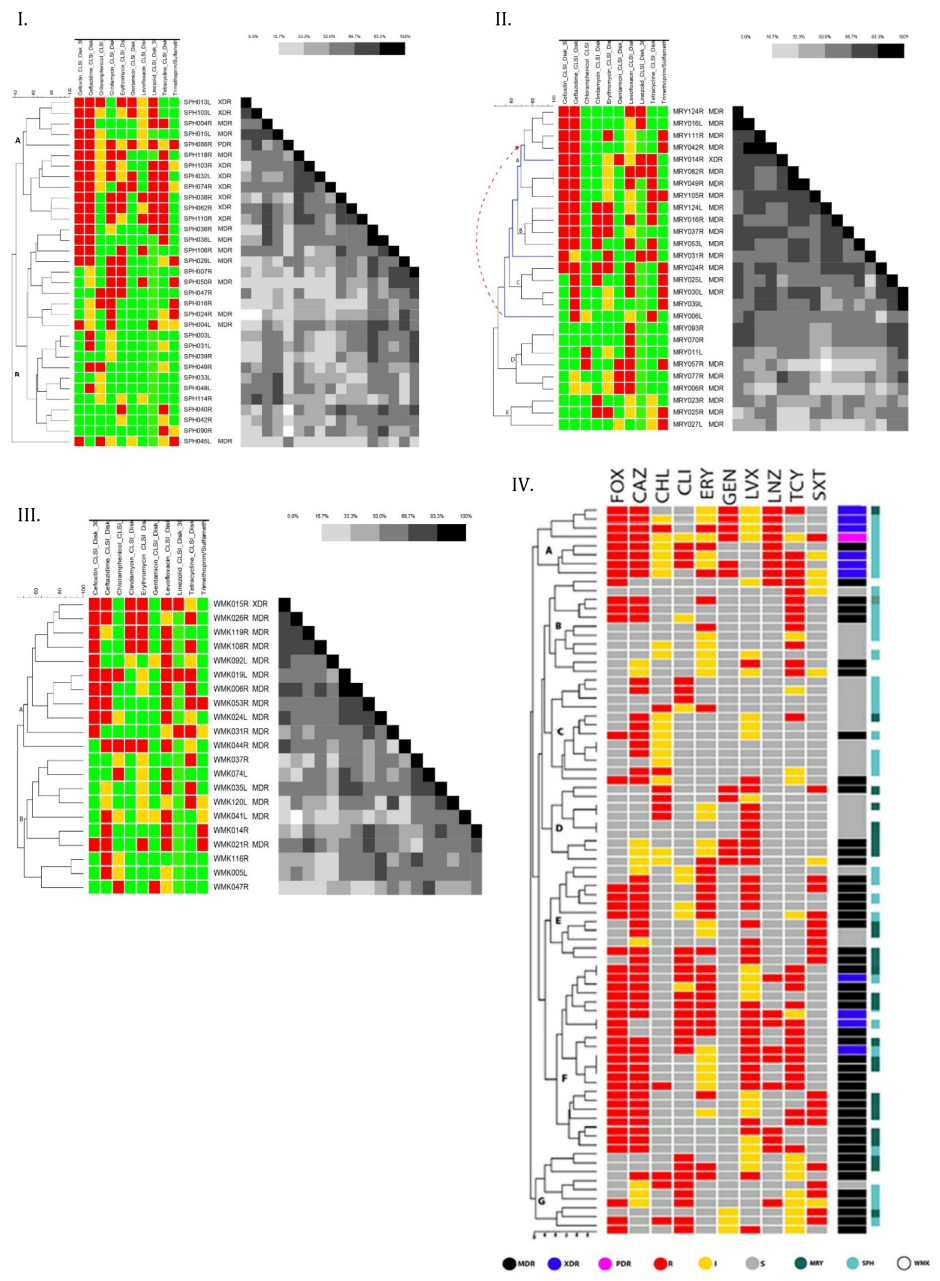
Dendrogram, constructed using BioNumerics™ software (version 7.6, Applied Maths) and the Dice coefficient, showing the overall antibiotic susceptibility profiles of *S. aureus* isolates across the three study centers. Scale bar indicates degree of similarity. MRY=Maryam Abacha Women’s and Children’s Hospital, SPH=Specialist Hospital Sokoto, WMK=Orthopaedic Hospital Wamakko, MDR=multidrug-resistance; XDR=extended drug-resistance, PDR=pan-drug-resistance, resistant (R, red), intermediate (I, yellow); susceptible (S, green/grey)

Figure 5 (I) (Fig. 5) shows a dendrogram-based cluster analysis of the susceptibility patterns of 33 *S. aureus* isolated from Specialist Hospital toward ten antibiotics. In the figure, red indicates resistance to the matching antimicrobials, yellow indicates intermediate susceptibility, and green indicates susceptibility. The profiles identified 19 multi-resistant strains of *S. aureus* (57.6%, n=19/33), out of which 52.6% (10/19) were MDR, 42.1% (8/19) were XDR, and 5.3% (1/19) were PDR. Cluster analysis identified two distinct clades (A and B) and one single-member cluster. The first clade (A) comprises most of the multiple resistance strains (12/19) defined by the WHO as high-priority strains, mainly based on their resistance to cefoxitin and linezolid with a similarity matrix of 16.7% to 100%. The majority of isolates in cluster B were susceptible to gentamycin (20/21) and exhibited complete to reduced susceptibility to linezolid and cefoxitin. The cluster includes 7/19 of multiple-resistant strains. The profiles of strains clustered in clade B are largely heterogeneous with a similarity matrix cover of 50% at most. The single-member cluster is the only multidrug-resistant strain in the study center that is susceptible to ceftazidime antibiotics. Of the 33 isolates, 54.5% (n=18.95% CI; 33.8–68.8) were resistant to cefoxitin, 36.4% (n=12, 95% CI; 21.0–54.9) to linezolid, 33.3% (n=11, 95% CI; 18.5–51.9) to erythromycin and 42.4% (n=14 95% CI; 25.9–60.6) to clindamycin. The majority of the isolates were resistant to ceftazidime (60.6%, n=20, 95% CI: 42.2–76.6). 

The susceptibility profiles of 27 *S. aureus* isolates of Maryam Abacha Women’s and Children’s Hospital were observed to group into five discriminatory clusters, as illustrated with a dendrogram (Figure 5 [II] (Fig. 5)). The clades A and B were clustered primarily based on high percentage resistance to cefoxitin and ceftazidime, clade C based on susceptibility to cefoxitin and resistance to ceftazidime, with a similarity matrix of 16.7% to 100%. Clades D and E were largely susceptible to cefoxitin and ceftazidime with a similarity matrix of 33.3% to 50%. The figure also uncovers a trend upstream from clade C to A in which each single-member sub-cluster in the clade acquires resistance to an additional antibiotic, from non-multidrug resistant to MDR and XDR. A total of twenty-two isolates (81.5%) demonstrated multi-resistance toward ten antibiotics tested in this study. Overall, the highest percentage of resistance was observed toward ceftazidime (63%, n=17, 95% CI; 35.7–74.0) and cefoxitin (51.9%, n=14, 95% CI; 32.4–70.9). About 25.9% (n=7, 95% CI; 11.9–46.6) of the *S. aureus* isolates were resistant to erythromycin, 29.6% (n=8, 95% CI; 14.5–50.3) to clindamycin, and 14.8% (n=4, 95% CI; 4.8–34.6) to gentamicin. Only 5 out of 27 isolates (18.5%, 95% CI; 7.0–38.7) were resistant to linezolid. Moreover, results indicated that most MDR isolates tended to exhibit resistance towards cephamycin (CEPHAM/second-generation) and third-generation cephalosporin (CEPH3) classes, i.e., a majority of isolates that were resistant (intermediate or complete) to ceftazidime (70%, n=14/20) were also resistant to cefoxitin. 

All multiple-resistant isolates make up 71.4% (n=15/21) of all confirmed *S. aureus* in Orthopedic Hospital Wamakko. Only three (14.3%, 95%CI; 3.8–37.4) out of 21 *S. aureus* strains were linezolid resistant. Resistance to cefoxitin was high at 52.4% (n=11/21, 95% CI; 30.4–73.6). The highest level of resistance was to ceftazidime (57.1%, n=12, 95% CI; 34.4–77.4). Low resistance to gentamicin (4.8%, 95%CI; 0.3–25.9) was observed. Resistance to erythromycin and clindamycin was 28.6% (n= 6/21, 95% CI; 12.2–52.3) and 23.8% (n=5, 95% CI; 9.1–47.5), respectively.

For the 21 *S. aureus* isolates of Orthopedic Hospital Wamakko identified in this study, a hierarchical clustering analysis based on their antimicrobial sensitivity patterns was computed to create a dendrogram using the Dice coefficient (Figure 5 [III] (Fig. 5)). The key component of the analysis is repeated calculation of similarities between isolate susceptibility patterns and between clusters once isolates begin to be grouped into clusters. The outcome is represented graphically as a dendrogram. The dendrogram produced two major clades (A and B). From the dendrogram it is clear that most of the multiple-resistant isolates (73.3%, n=11/15) were clustered in one major clade (A) and the rest into clade B. The first clade (A) houses mostly cefoxitin, ceftazidime and levofloxacin resistant isolates. Clade B comprises 26.7% (n=4/15) of multiple-resistant isolates. All multiple-resistant isolates make up 71.4% (n=15/21) of all confirmed *S. aureus*. Of these, only one isolate was extremely drug resistant. The data indicate that the multiple-resistant isolates form a more coherent cluster than others isolates (with a similarity gradient of 50% to 100%). Figure 5 (Fig. 5) also depicts the susceptibility profiles of isolates from Orthopaedic Hospital Wamakko. Of the 21 *S. aureus* strains, only three (14.3%, 95% CI; 3.8–37.4) were linezolid resistant. Resistance to cefoxitin was high at 52.4% (n=11/21, 95% CI; 30.4–73.6). The highest level of resistance found was to ceftazidime (57.1%, n=12, 95% CI; 34.4–77.4). Low resistance to gentamicin (4.8%, 95% CI; 0.3–25.9) was observed. Resistance to erythromycin and clindamycin were 28.6% (n=6/21, 95% CI; 12.2–52.3) and 23.8% (n=5, 95% CI; 9.1–47.5), respectively.

Antibiotic resistance profiles of all *S. aureus* isolates are clustered in a dendrogram shown in Figure 5 (IV) (Fig. 5). Eighty-one strains were clustered into seven clades (A to G) visible in terms of their similarities. The graph also shows a total of 43 MDR, 10 XDR, one (1) PDR. 

Clade A is a cluster of 8 strains, most of which are extremely drug resistance (6/8) at 90% similarity. It houses the only PDR strain in the study. Members of this clade were resistant to a minimum of 5 antibiotic classes and a maximum 10 classes. Most of the isolates in the cluster were isolates from Specialist Hospital Sokoto. At 60% similarity, clade B comprises 5 MDR strains that are resistant to 5 out of 10 antibiotic classes used in this study (FOX-CAZ-ERY-LVX-TCY). The strains in clade C are predominantly resistant to 3 antibiotic classes (CAZ-CHL-LVX). It houses 2 MDR strains. Clade D houses 4 MDR and are predominantly resistant to 2 to 5 classes (CAZ-CHL-ERY-GEN-LVX). Clade E contains 8 MDR that are chiefly resistant to five classes (FOX-CAZ-ERY-LVX-SXT). Clade F is the largest cluster, as it houses 17 MDR and 4 XDR at 80% similarity. Isolates in this clade are mostly resistant to 6 classes of antibiotics profiled as FOX-CAZ-ERY-LVX -LNZ-TCY. Finally, clade G houses 8 MDR strains with a resistance profile of CAZ-CLI-GEN-LVX-TCY-SXT.

The overall susceptibility of the 81* S. aureus* isolates is shown in Table 4 (Tab. 4). Resistance to ceftazidime was highest (60.5%, n=49.95% CI; 55.3–76.5) among the isolates, followed by cefoxitin (53.1%, n=43, 95%CI; 41.7–64.2). Resistance to erythromycin, clindamycin and linezolid was shown for 30.9% (n=25, 95% CI; 20.2–40.9), 21% (n=24, 95% CI; 13.0–31.8) and 23.5% (n=19, 95% CI; 16.1–35.8) of the isolates, respectively. In contrast, only 11 (13.6) of the isolates were found to be resistant to both gentamicin and chloramphenicol (95% CI; 7.3–23.4).

**Figure 6:**
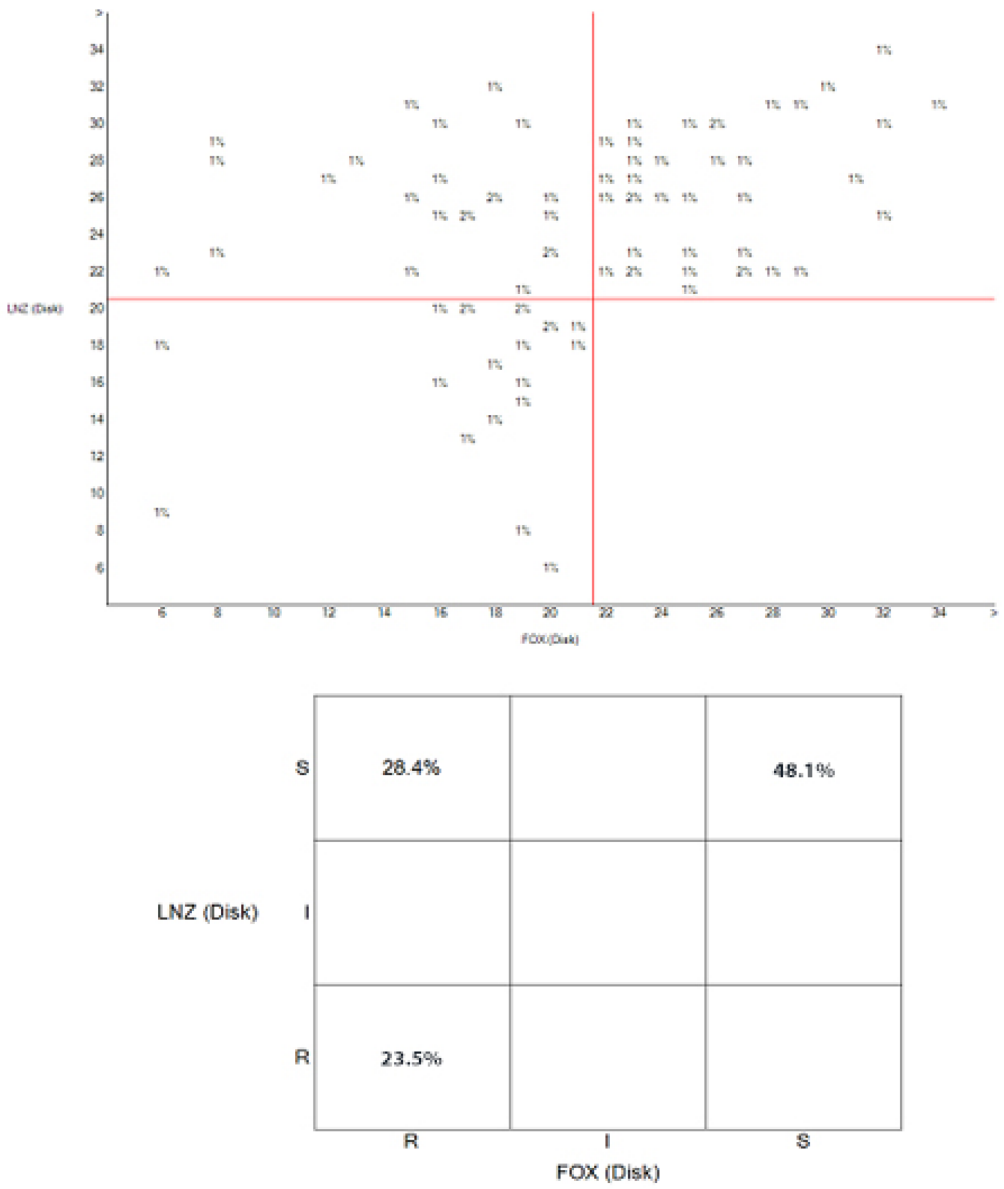
Scatterplot showing the relationship between cefoxitin (FOX) and linezolid (LNZ) susceptibility profiles of *S. aureus* isolates

The relationship between the zones of inhibition of cefoxitin and linezolid is depicted in the scatterplot in Figure 6 (Fig. 6), which shows that 23.5% of isolates were resistant to both linezolid and cefoxitin, 28.4% were linezolid susceptible and cefoxitin resistant, and 48.1% of the isolates were susceptible to both linezolid and cefoxitin. The unpopulated area of the plot reflects the fact that there is no cefoxitin-susceptible isolate that was linezolid resistant.

The doughnut chart in Figure 4A (Fig. 4) displays the overall prevalence of *S. aureus* (61.8%) after phenotypic characterization. As illustrated in Figure 4B (Fig. 4), the percentage of *S. aureus* that is MRSA is 46.9% (38/81). The figure also shows the distribution of MRSA among the study centers.

The relationships between MRSA and associated risk factors are shown in Table 5 (Tab. 5). The highest percentage of MRSA colonization occurred at Specialist Hospital Sokoto (39.5%, n=15, 95%CI; 8.4–24.7). The right nostril harbored more MRSA, with a prevalence rate of 65.8% (n=25, 95% CI; 16.2–36.9), than did the left nostril. Among participants, healthcare workers harbored more MRSA 39.5% (n=15) compared with other participants. Nasal carriage rate was also the highest among female participants (65.8%, n=25, 95% CI; 16.2–36.9). Participants who belonged to the age group 21-25 years had the highest carriage rate (36.8%, n=14, 95% CI; 7.7–23.5). In general, 86.8% (n=33, 95% CI; 22.7–46.3) of the participants who were MRSA carriers had taken antibiotics prior to the examination (2 months). Nasal colonization with MRSA was observed more in participants without HIV/AIDS (73.7%, n=28, 95% CI), with diabetes (55.3%, n= 21, 95% CI; 13.0–32.1), with a history of tobacco use (57.9%, n=22, 95% CI; 13.8–33.3) and without renal disease (89.5%, n=34, 95% CI; 23.5–47.5). 

**Figure 7:**
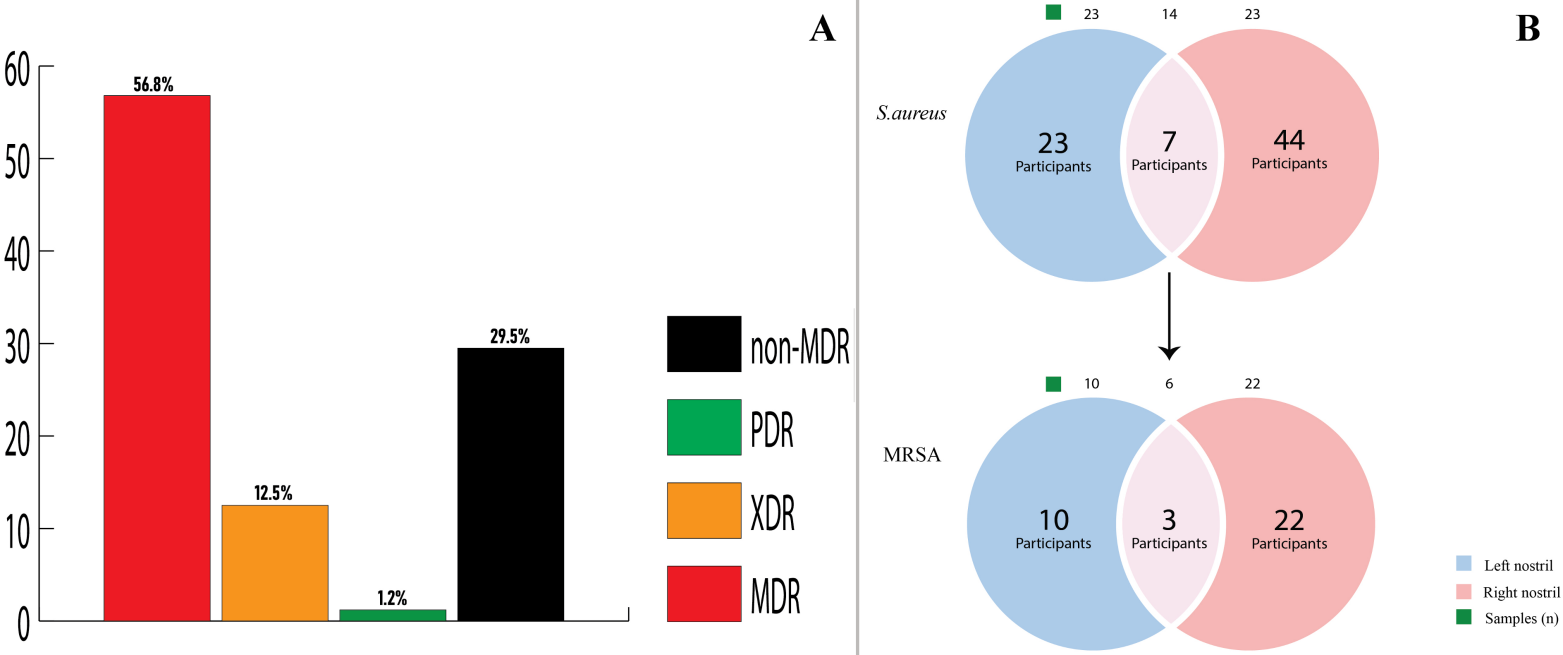
(A) Bar graph of the percentage distribution of multidrug-resistant *Staphylococcus aureus* (MDR), extended-drug-resistant *S. aureus* (XDR), pan-drug-resistant *S. aureus* (PDR) and non-multidrug-resistant *Staphylococcus aureus* (non-MDR); (B) Venn diagram of nasal distribution patterns of *S. aureus* and MRSA among participants; blue/pink sections and intersections represent the number of participants with left, right and dual nostril carriages, respectively

The bar graph in Figure 7A (Fig. 7) shows the individual percentage distribution of multidrug-resistant *Staphylococcus aureus* (MDRSA), methicillin-susceptible *S. aureus* (MSSA), methicillin-resistant *S. aureus* (MRSA), extreme-drug-resistant *S. aureus* (XDRSA) and pan-drug-resistant *S. aureus* (PDRSA). 

The relative colonization rates of MRSA and the nasal distribution patterns of *S. aureus* and MRSA among participants with respect to left vs right nostrils or both is shown in the Venn diagram in Figure 7B (Fig. 7) . Out of 74 participants, 7 (9.4) were dual-nostril carriers of *S. aureus*, out of which 3 (42.9%) were MRSA carriers. Table 6 (Tab. 6) shows that out of 116 participants who harboured coagulase positive *S. aureus*, 16 (13.7%) were dual-nostril carriers. The results for the multivariate analysis of demographic and risk factors for Staphylococcus aureus and MRSA nasal colonization is presented in Table 7 (Tab. 7).

## Discussion

Methicillin-resistant *Staphylococcus aureus* (MRSA) is a major human pathogen and a leading cause of nosocomial infections [8]. Recommendation in hospital surveillance protocols disagree as to whether both nostrils should be sampled, and those that favor the sampling of both nostrils habitually recommend the use of a single swab [23]. Implicit in such protocols is the idea that nostrils are homogenous regarding the bacterial populations they carry, thus justifying treating two separate nostrils as if they were a single body site. This study tested this premise by sampling right and left nostrils separately for *S. aureus* carriage.

This study focused on *S. aureus* and MRSA isolates from the anterior nares of participants from three hospitals in Sokoto state. We documented carriage rates of 61.8% and 46.9% for *S. aureus *and MRSA in this study. These observed rates are consistent with the burden of S*. aureus* being higher and MRSA being lower than 50% prevalence in several African studies [3], [4], [5]. These reported rates may not be unconnected to non-adherence to drug prescription, self-medication, and poor hygiene practices coupled with suboptimal sanitation and water supply facilities in African countries [3], [24], [25].

Among the study centers, Specialist Hospital Sokoto (SPH) had the highest *S. aureus* prevalence of 40.7% (n=33, 95% CI: 22.7–46.3). This center recorded the highest percentage of MRSA colonization (39.5%, n=15, 95% CI; 8.4–24.7). This may be because the hospital serves numerous patients from within and outside the metropolis, including referred patients from the other two health facilities. Epidemiologically, hospitals are reported to be connected by the patients they share, and their degree of connectedness influences the rates of spread of hospital-acquired bacteria [26].

Overall, the category of participants with the highest carriage rate was healthcare workers, probably because of greater exposure to circulating microbes [27]. Healthcare workers (HCWs) are more often colonized, serving as a reservoir for endogenous infections and dissemination [28]. This is corroborated by the 31% prevalence rate reported among health-care workers by other authors [29]. 

Sixty-three percent (63%; 95%, CI: 37.7–57.1) participants in this study were cultured-positive for *S. aureus* in the right nostril compared to 37% (95%, CI: 20.2–42.8) in the left nostril. In total, 74 participants carried 81 *S. aureus* in their nostrils, out of which only 7 (9.5%, n=7/74) participants were dual-nostril carriers, and in turn, only 3 (42.9%, n=3/7) of the latter were dual carriers for MRSA. Kildow et al. [30] reported that nasal carriers of *S. aureus* were 60% likely to harbour the bacteria in a single nostril than in both (test of population proportions, *P*=0.0015). The participants in this study were more likely to carry more *S. aureus* in the right nostril than in the left (P=0.0010). In terms of MRSA, a significant association (P=0.0062) was recorded between right- and left-nostril carriage. Dual-nostril (MRSA) carriage, however, was less probable among participants than was single-nostril carriage (P=0.0001). This may be attributable to cyclic events known as the nasal cycle, where nasal airflow is greater in one nostril than in the other due to transient asymmetric nasal passage obstruction by swollen tissue. This physically blocks the passage of air in one nostril more than in the other, resulting in an increase in the filling pressure and other pathophysiological (e.g., nasal congestion) phenomena which favor higher bacterial adherence to the nasal epithelium [31].

In this study, the percentage carriage of *S. aureus* and MRSA was higher among the female participants. Female sex hormones (estrogen) have been related to high nasal *S. aureus* carriage. Over the past two decades, studies [32] have related high estrogen levels as the impetus for a shift in *S. aureus* carriage status. High estrogen levels could have predisposed the participants to higher colonization by altering the properties of nasal surfaces that serve as barriers to infection (e.g., local mucosal atrophy and decreased mucin secretion). This trend agrees with the findings of Liu et al. [32].

The rate of *S. aureus* carriage was higher among married participants (50.6%; 95% CI: 36.3–68.7) than unmarried participants (49.4%; 95% CI: 35.3–67.3). Similarly, the rate of MRSA colonization was also higher among married participants (65.8%; n=25/38, 95% CI, 42.6–97.1). This rate is in agreement with the work of Abiye et al. [33] that showed a significant association of MRSA colonization with marital status, i.e. married participants were more frequently colonized than unmarried ones. Frequent contacts with spouses, family members and friends may have facilitated this occurrence [34].

This study also showed that 86.8% (n=33, 95% CI; 22.7–46.3) and 80.2% (95% CI: 50.2–82.9) of the participants who were MRSA-colonized and *S. aureus* carriers, respectively, had taken antibiotics prior to testing (2 months). In addition to the high probability of *S. aureus* becoming resistant to antibiotics after prior antibiotic exposure, factors such as abuse or misuse of antimicrobial agents in addition to surgical procedures that disturb the mucocutaneous barriers may collectively contribute to a decrease in patient’s resistance to invading bacteria, with subsequently increased risk of antibiotic-resistant staphylococcal infection. This result agrees with the study by Ansari et al. [35]. 

In terms of age, the highest prevalence rates occurred among the age group of 21–25 (34.6% [95% CI: 18.6–40.4]). This may be related to the fact that participants in this age groups are active and in contact with large groups of people at work, school and other crowded environments. The results are corroborated by Solomon et al. [36]. 

This study also revealed a lower prevalence rate of *S. aureus* colonization among participants who were diagnosed as HIV positive (18.5% [95% CI: 7.0–38.7]). Nasal colonization with MRSA was more frequently observed in participants without HIV/AIDS (73.7%, n=28,); this finding was in agreement with the study by Befus et al. [37]. HIV-infected patients are at greater risk (poor immunity, exposure to antibiotics from recent hospitalizations and earlier MRSA infection or colonization) of MRSA colonization relative to the general population [38], [39]. Nevertheless, it was interesting that nasal colonization with MRSA was more often found in respondents without HIV/AIDS in our study. In fact, reduced MRSA colonization has been reported among HIV/AIDS patients on highly active antiretroviral therapy (HAART) by some studies [40], [41]. This unintended effect of antiretroviral drugs could help explain our findings. The reduced MRSA colonization may be linked to non-selection of drug-resistant microorganisms subsequent to reduced antibiotic usage among patients on HAART treatment [42]. Furthermore, a decreased HIV disease progression in patients undergoing therapy may have led to a reduced frequency of *S. aureus* and MRSA colonization [39], [43]. 

Regarding tobacco use, this study found that the colonization rate of *S. aureus* was higher among non-users of tobacco (60.5% [95% CI: 36.2–64.8]). Smokers’ susceptibility to respiratory tract infections is attributed to an altered immune status and other deleterious effects of cigarette smoke components, such as damage to the airways which could eventually result in bronchitis [44]. Earlier studies have found high carriage rates of *S. aureus* and MRSA among non-smokers [24], [45], [46]. In our study participants, smoking had a protective effect against *S. aureus* colonization and established a nasal environment that impedes their growth.

This study also showed that participants with diabetes and renal disease did not have a high prevalence rate of *S. aureus* carriage (44.4% [95% CI: 25.2–49.8]) and (6.2% [95% CI: 1.6–11.6]), respectively, when compared with participants who were not subject to these factors. Immune and lung dysfunction commonly associated with diabetes may lead to a worse response to antibiotic treatment and thus increased carriage of MRSA and *S. aureus* carriage among these patients compared with non-diabetic patients. This agrees with the works of several other authors [47], [48], [49].

By univariate analysis, this study proved that several factors were associated with an increased risk of acquiring *S. aureus*: hospital, nostril, participant, gender, marital status, age group, previous antibiotic use, HIV status, diabetes, tobacco usage and renal disease. However, after controlling for the confounding effect of these variables by means of multivariate regression, only two variables (HIV and diabetes status) remained significantly associated with *S. aureus* acquisition (P≤0.005). In terms of MRSA, binary logistic regression analysis associated five factors (tobacco usage, HIV status, diabetes, marital status and nostril) as statistically significant contributors to nosocomial MRSA acquisition. In corroboration with some earlier studies [33], [50], we could not statistically associate gender and previous antibiotic [51] usage as risk factors for *S. aureus* and MRSA colonization. The importance of antibiotic use as a risk factor is increased by a prolonged period from admission to isolation of MRSA, as it increases the time for exogenous acquisition antibiotic resistant strains [51]. The significance of antibiotic usage as a risk factor may have been underestimated in this study because we were unable to document the duration of respondents’ hospitalization. 

A majority of the isolates were resistant to ceftazidime (60.6%, n=20, 95%CI: 42.2–76.6). The susceptibility profile identified 19 multi-resistant strains of *S. aureus* (57.6%, 19/33), of which 52.6% (10/19) were MDR, 42.1% (8/19) XDR, and 5.3% (1/19) were PDR. Most (12/19) of these were clustered in a group with 16.7% to 100% similarity. This may reflect the fact that the isolates originated from an environment where most of these antibiotics are in use. Multidrug resistance is probably an indicator that a very large proportion of bacterial isolates have been exposed to several antibiotics [52].

A total of 22 isolates (81.5%) demonstrated multi-resistance toward ten antibiotics tested in Maryam Abacha Women’s and Children’s Hospital. In general, the highest percentage of resistance observed was to ceftazidime (63%, n= 17, 95% CI; 35.7-74.0) and cefoxitin (51.9%, n=14, 95% CI; 32.4–70.9). About 25.9% (n=7, 95% CI; 11.9–46.6) of the *S. aureus* isolates were resistant to erythromycin, 29.6% (n=8, 95% CI; 14.5–50.3) to clindamycin and 14.8% (n=4, 95% CI; 4.8–34.6) to gentamicin. Only 5 out of 27 isolates (18.5%, 95% CI; 7.0–38.7) were resistant to linezolid. Moreover, results indicated that most MDR isolates tended to exhibit resistant towards antimicrobial agents of classes CEPHAM and CEPH3, i.e., the majority of isolates resistant (intermediate or complete) to ceftazidime (70%, n=14/20) were also resistant to cefoxitin. Several reasons have been advanced to explain why hospital isolates are resistant to multiple antibiotics. This includes the intrinsic nature of some isolates to be resistant to the drug, either due to lack of the target site for that drug, inability of the drug to transit through the organism cell wall or membrane and reach its site of action, acquisition of plasmids which is most common among hospital isolates, drug efflux and target site modification or permeability [53].

Ceftazidime was the most prevalent isolate (60.5%, n=49, 95% CI; 55.3–76.5), followed by cefoxitin (53.1%, n=43, 95% CI; 41.7–64.2). Gentamycin is the only drug in this study that was most effective against *S. aureus*. One reason for its effectiveness might be associated with the fact that gentamycin is infrequently used, as it administered by injection. This form of administration is far less amenable to self-medication than orally administered antibiotics [54]. Reports have documented that resistance to cefoxitin shown by disk diffusion can be used for MRSA strain detection in routine testing, because cefoxitin is regarded as a potential inducer of the system that regulates *mecA* gene [55]. For this reason, isolates which were found to be resistant to cefoxitin were also considered methicillin resistant.

Overall, linezolid was effective (76.5%) across the study centers, although its resistance percentage was still 23.5% (95%CI; 16.1–35.8). Its effectiveness may be connected to the fact that, as opposed to the other antibiotics used in this study, linezolid is not routinely prescribed or administered. Conversely, resistance to linezolid observed in this study may have been mediated through ribosomal mutations (23S*rRNA*) or methylation of 23S*rRNA* by the horizontally transferred *Cfr* plasmid-borne ribosomal methyltransferase [56].

The increased incidence of multi-resistant strains observed in this study may be due to the included study centres being secondary healthcare centres, and patients from adjoining districts and even villages are admitted for treatment. Before admission to the hospital, most of the participants may have taken different antibiotics prescribed by general practitioners or purchased over-the-counter, often in improper doses [57].

There are several limitations to this study. First, molecular differentiation of the strains into community and hospital-acquired (CA and HA) strains was not carried out to further determine the directionality of their spread. Second, the use of nasal cultures solely for *S. aureus* and MRSA detection has been reported to have a sensitivity of 78% to 85% which is much higher than cultures from other body sites [58], [59]. The sensitivity of our sampling technique was not accounted for during sample size determination. Thus, our reported rates may not give a full account of the situation in the region. Nevertheless, our results revealed that MRSA colonization among the studied respondents occurred.

## Conclusions

This study found a prevalence of 61.8% and 46.9% of *S. aureus* and MRSA among the studied hospitals in Sokoto state, indicating that the nares of the hospital populace are not free from *S. aureus* and MRSA colonization. Especially the fact that MRSA colonizes nostrils of participants (associated with risk factors HIV, marital status, diabetes, and tobacco usage) did not occur by chance. In total, 74 participants carried 81 *S. aureus* in their nostrils, out of which only 7 (9.5%, n=7/74) participants were dual-nostril carriers. Among these, only 3 (42.9%, n=3/7) were dual carriers for MRSA. Nasal carriers of *S. aureus* were significantly more likely to carry in one nostril than in both (test of population proportions, P=0.0001). A majority of the isolates were resistant to multiple antibiotics. Resistance was predominantly to ceftazidime and cefoxitin. In light of the findings in this study, we recommend that nasal swabs should be taken from the right and left nostrils of patients using two different commercially available swab sticks, as carriage in both nostrils is not homogeneous, i.e., each nostril should be treated as a separate anatomical site to avoid underestimation of MRSA carriage. Another method of swabbing both nostrils with one swab stick starting from the left may also prove effective in determining MRSA carrier status of respondents. We need to update our healthcare workers, especially physicians, microbiologists, infection control practitioners and bureaucrats managing health care about MRSA emergence and spread.

## Notes

### Competing interests

The authors declare that they have no competing interests.

### Acknowledgments

We would like to express our profound gratitude to Mr. B.S. Abdulmalik and Mrs. Halima Salihu of Usmanu Danfodiyo University Sokoto, Nigeria for their contributions.

## Figures and Tables

**Table 1:**
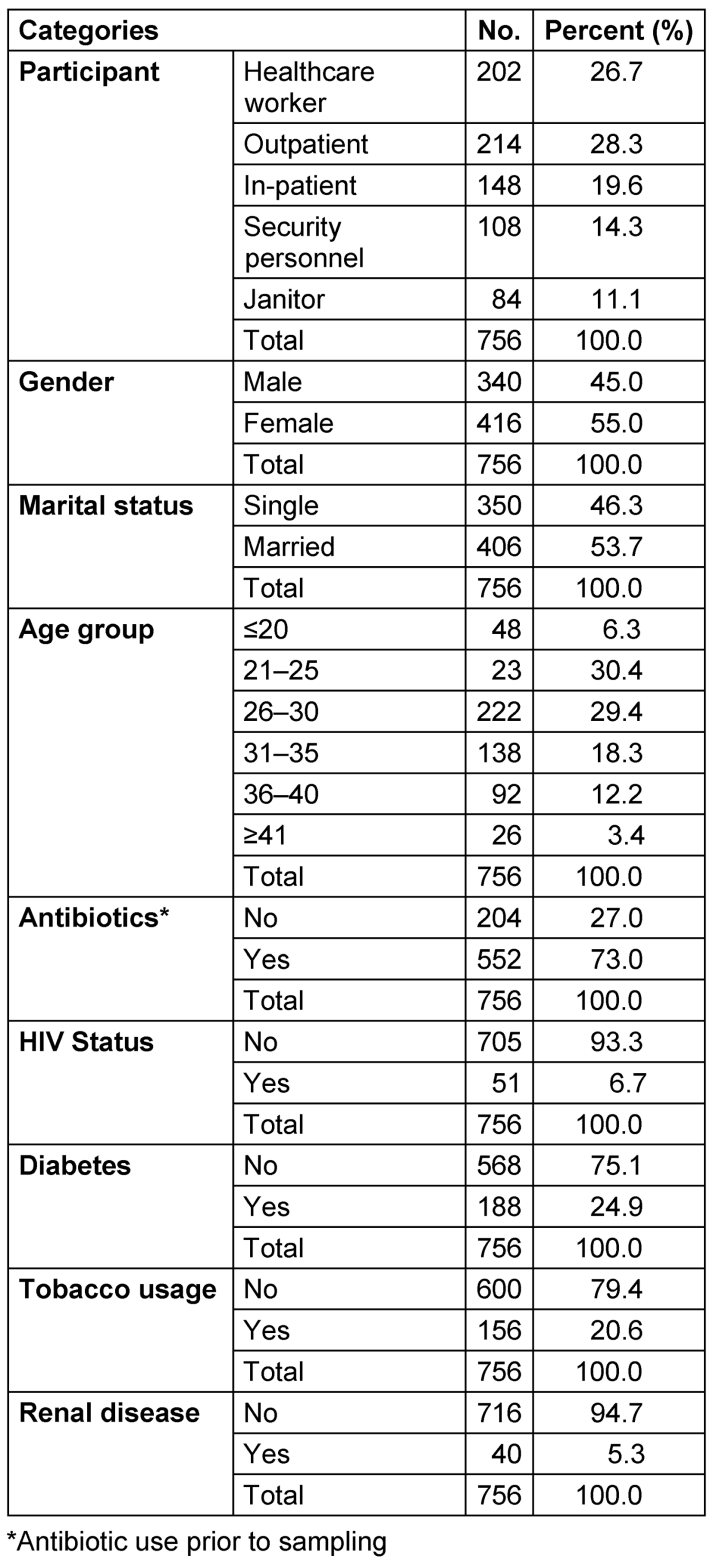
Summary of demographics of study participants

**Table 2:**
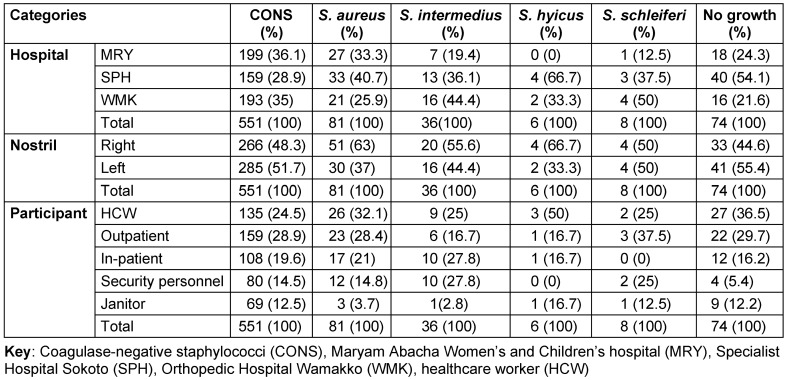
Distribution of coagulase-negative and coagulase-positive staphylococcal isolates

**Table 3:**
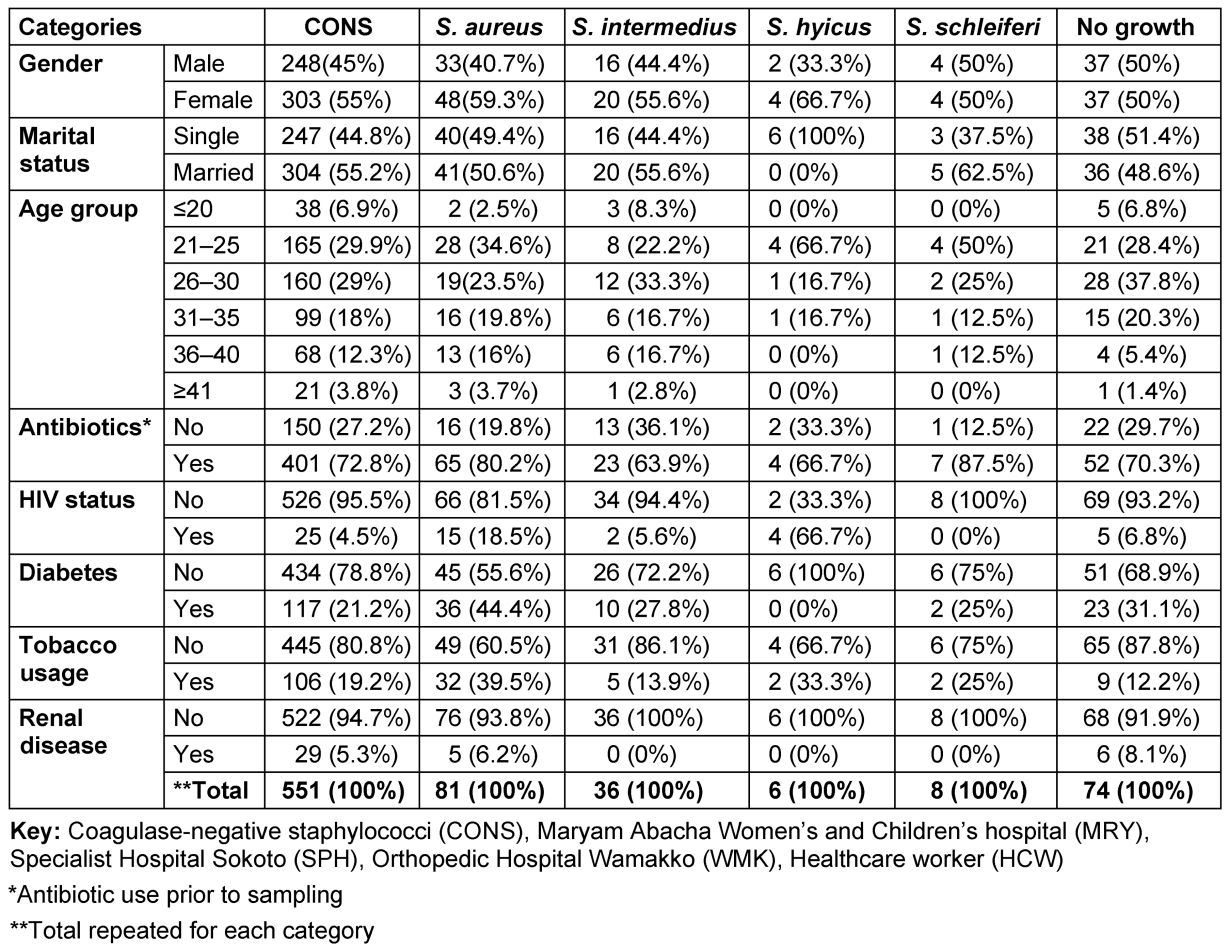
Distribution of coagulase-negative and coagulase-positive staphylococcal isolates by risk factor

**Table 4:**
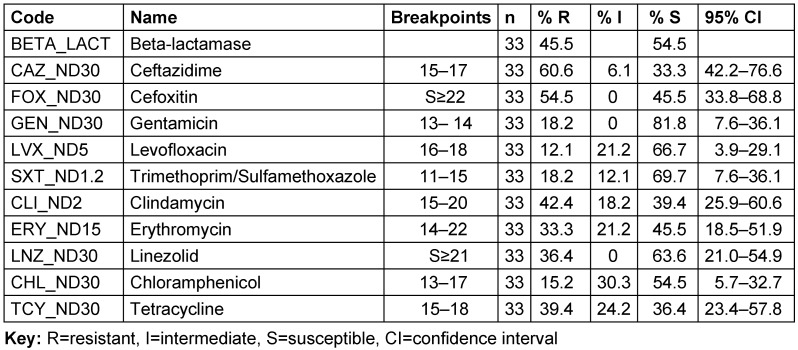
Antibiotic susceptibility profiles of *S. aureus* isolates from Specialist Hospital Sokoto

**Table 5:**
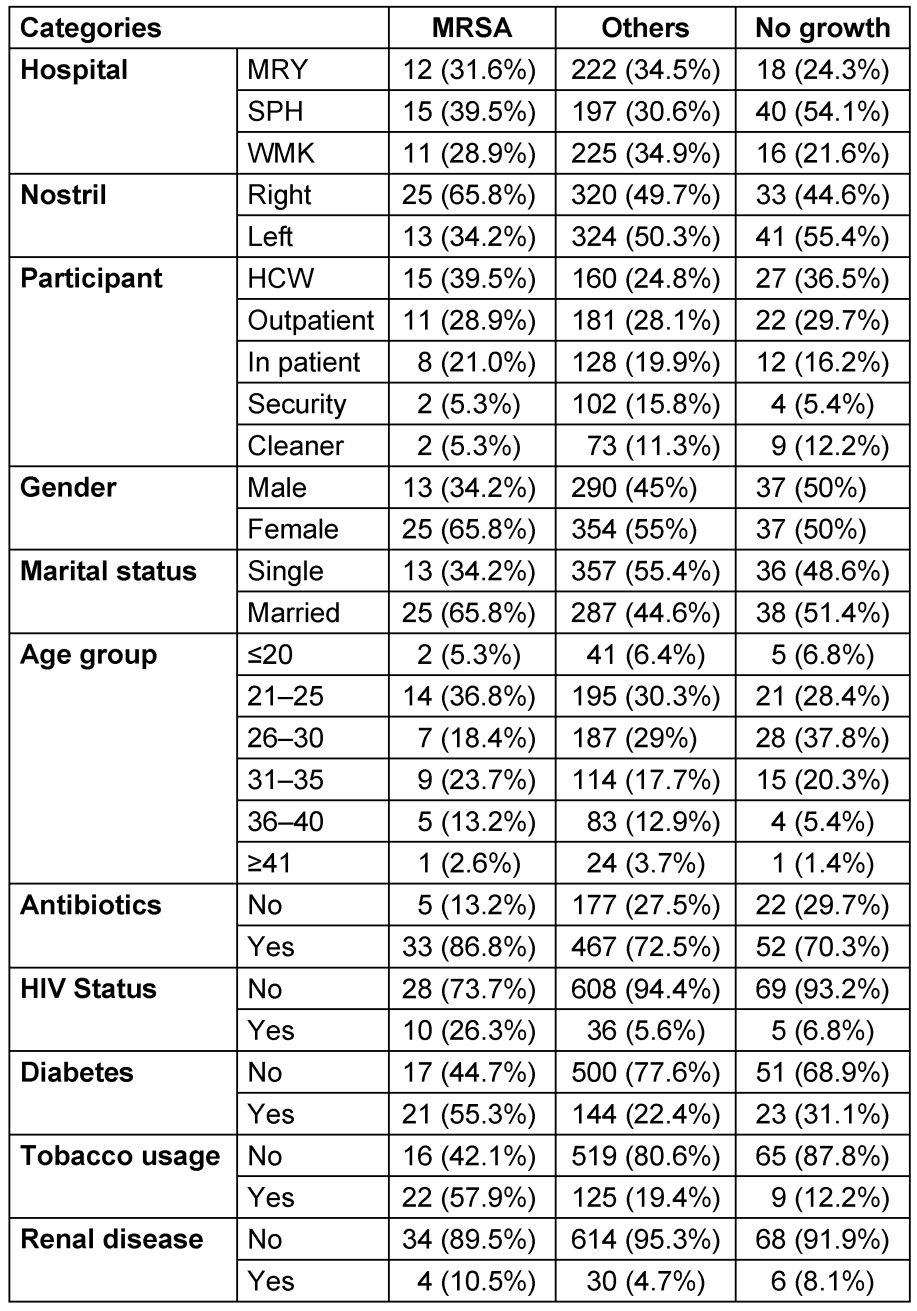
Distribution of MRSA isolates

**Table 6:**
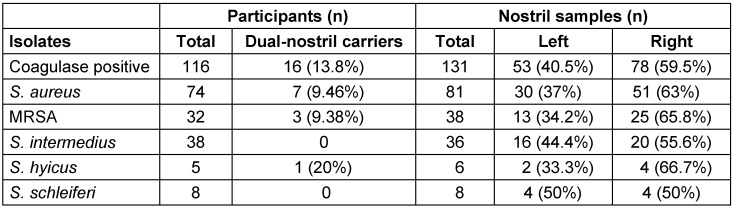
Distribution of staphylococcal isolates across participants and nostril samples

**Table 7:**
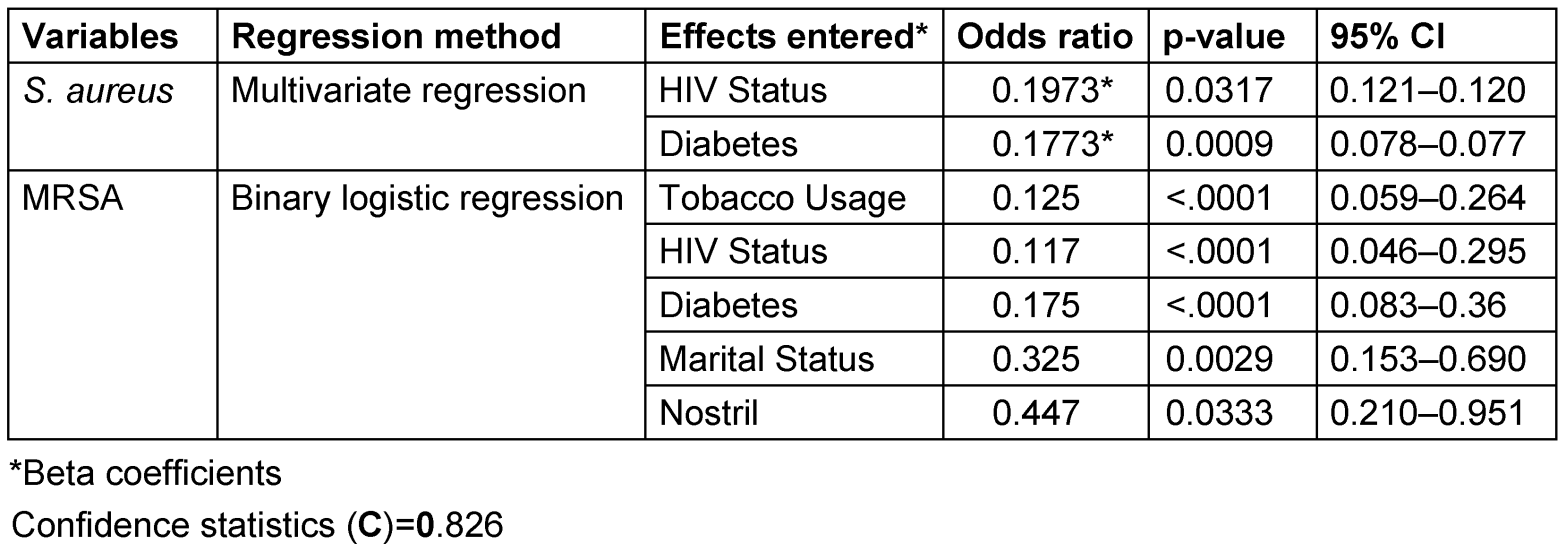
Analysis of demographic and risk factors for *Staphylococcus aureus* and MRSA nasal colonization
